# Stem cell-based therapy for systemic sclerosis

**DOI:** 10.1093/rap/rkad101

**Published:** 2023-11-20

**Authors:** Maryam Zare Moghaddam, Mohammad Javad Mousavi, Somayeh Ghotloo

**Affiliations:** Department of Immunology, Shahid Sadoughi University of Medical Sciences, Yazd, Iran; Department of Hematology, Faculty of Allied Medicine, Bushehr University of Medical Sciences, Bushehr, Iran; Autoimmune Diseases Research Center, Kashan University of Medical Sciences, Kashan, I.R. Iran; Department of Clinical Laboratory Sciences, School of Allied Medical Sciences, Kashan University of Medical Sciences, Kashan, Iran

**Keywords:** rheumatic autoimmune disorders, stem cell, systemic sclerosis, therapy

## Abstract

Autoimmune diseases, including SSc, are prevalent, affecting autologous connective tissues and caused by the breakdown of self-tolerance mechanisms of the immune system. During the last 2 decades, stem cell therapy has been increasingly considered as a therapeutic option in various diseases, including Parkinson’s disease, Alzheimer’s disease, stroke, spinal cord injury, multiple sclerosis, inflammatory bowel disease, liver disease, diabetes, heart disease, bone disease, renal disease, respiratory disease and haematological abnormalities such as anaemia. This is due to the unique properties of stem cells that both divide and differentiate to the specialized cells in the damaged tissue. Moreover, they impose immunomodulatory properties affecting the diseases caused by immunological abnormalities such as SSc. In the present review, the efficacy of stem cell therapy with two main types of stem cells, including mesenchymal stem cells and hematopoietic stem cells, will be reviewed. Moreover, other related issues, including safety, changes in immunological parameters, suitable choice of stem cell origin, conditioning regimen and complications of stem cell treatment will be discussed.

Key messages

- SSc is an autoimmune disease with abnormal collagen deposition in the skin and internal organs.
- Mesenchymal and haematopoietic stem cells improve disease activity and severity.
- Both treatments result in an improvement in the skewed immunological parameters in favour of disease improvement.
- Both mesenchymal and haematopoietic stem cells show safety.

## Introduction

In the last 2 decades, stem cell therapy (SCT) has increased due to the unique properties of these cells. After dividing, a stem cell generates a new stem cell and differentiates to a specialized cell. Moreover, it has been shown that stem cell therapy is a safe, therapeutic method [1, 2].

There are several types of stem cells, including mesenchymal stem cells (MSCs) and haematopoietic stem cells (HSCs). Herein, MSC transplantation (MSCT) and HSC transplantation (HSCT) in SSc will be reviewed [3].

## SSc

SSc is a disease with abnormal collagen deposition in the skin and internal organs resulting in the fibrosis of these tissues as well as vasculopathy due to insufficient angiogenesis. Limited and diffuse cutaneous forms are the two main clinical subsets and can be distinguished by the extent of skin involvement, their autoantibody profile and the pattern of organ involvement. Few therapeutic options and a high rate of mortality make it difficult to deal with the disease. Although the exact pathogenesis remains unknown, predominant T cells activation, the existence of specific autoantibodies and the efficacy of the immunosuppressive drugs in the disease suggest breakdown of tolerance in the immune system could be involved in the disease pathogenesis. Many inflammatory cytokines and growth factors, including TGF-β, endothelin-1, IL-17, IL-23 and TNF-α, are correlated with the onset and progression of fibrosis [4, 5].

Treatments in SSc, including for early diffuse cutaneous SSc (dcSSc), and the use of organ-specific therapies have improved. Treatments for early dcSSc include immunosuppressive agents such as MMF, MTX, CYC, rituximab and tocilizumab [6]. However, standard therapy with CYC, either daily oral consumption or monthly i.v. injection, in SSc patients has demonstrated limited efficacy [7].

Patients with rapidly progressive early dcSSc might be eligible for autologous HSCT, which can improve survival. Morbidity from interstitial lung disease (ILD) and pulmonary arterial hypertension (PAH) is improving with the use of proven therapies. MMF has surpassed CYC as the initial treatment for SSc-ILD. Nintedanib (tyrosine kinase inhibitor), and possibly perfinidone, can be considered in SSc pulmonary fibrosis. PAH is frequently treated with initial combination therapy (e.g. with phosphodiesterase 5 inhibitors and endothelin receptor antagonists) and, if necessary, the addition of a prostacyclin analogue. RP and digital ulcers are treated with dihydropyridine calcium channel blockers (especially nifedipine), then phosphodiesterase 5 inhibitors or i.v. iloprost. Bosentan can reduce the development of new digital ulcers [6].

Trial data for other manifestations are mostly lacking. Research is needed to develop targeted and highly effective treatments, best practices for organ-specific screening and early intervention and sensitive outcome measurements [6].

**Figure 1.**
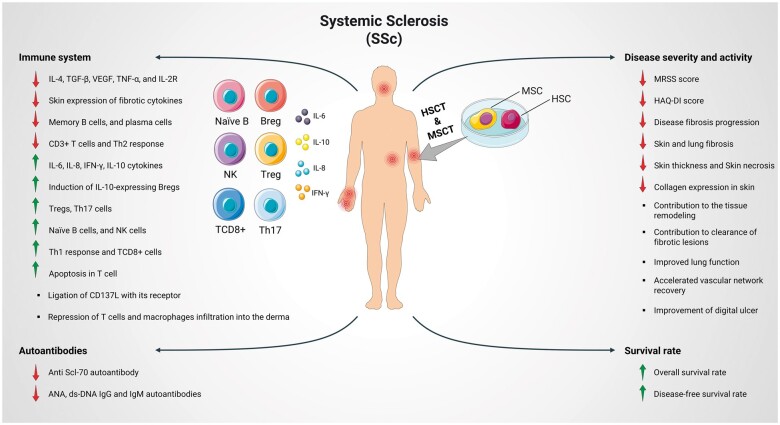
Effects of MSCT and HSCT on immunological and clinical parameters in SSc patients. A summary is presented of changes in the immunological parameters of SSc patients after MSCT and HSCT (left side of the figure). Moreover, changes in the severity, activity and survival rate of SSc patients are shown after treatment (right side of the figure)

The success of the first HSCT in SSc patients provided a platform for stem cell–based therapies in these patients [8, 9]. Results of SCT, including MSCT and HSCT, in SSc patients will be reviewed in the next sections (Table 1, Fig. 1).

**Table 1.** Results of SCT in SSc animal models and human patients in different studies

Type of stem cells	Results of SCT	Animal model/human	Reference
Combination of allogeneic MSCs and plasmapheresis	Improvement in mRSS Improvement in the lung function Decrease in anti-Scl-70 autoantibody titre Decrease of serum TGF-β and VEGF	SSc patients	[10]
MSCs	Improvement in the clinical course of the disease Improvement in the mRSS and HAQ-DI score	SSc patients	[10, 11]
Allogeneic MSCs	Improvement in the skin and pain score Ligation of CD137L with its receptor resulting in the induction of type 1 cell-mediated immune responses	SSc patients	[12]
Autologous MSCs	Accelerated vascular network recovery Restoration of blood flow Reduction of skin necrosis	SSc patient with critical limb ischaemia	[13]
Allogeneic MSCs	Improvement of digital ulcers Vasculoregenerative features Immunomodulatory properties	SSc patients	[14]
HSCs	Survival of 96.2% of patients after 5 years and 84.8% after 7 years of HSCT Event-free survival of 64.3% at 5 years and 57.1% at 7 years after HSCT	SSc patients	[15]
HSCs	Good response of 82% of patients in long-term follow-up	SSc patients	[15]
HSCs and CYC	Survival of 93% at both 5 and 10 years after HSCT Event-free survival rate of 50% at 5 years and 40% at 10 years after HSCT	SSc patients	[16]
HSCs and high-dose CYC	Acceptable survival rate Long-term event-free survival	Patients with early dcSSc	[17]
HSCs	Survival and disease-free survival rates of 90% and 70%, respectively, after 25.5 months of HSCT	SSc patients	[18]
HSCs	Skin improvement Significant decrease in the mRSS	SSc patients	[19]
PB-SCT and high-dose CYC	Significant decrease in skin involvement score Decrease in dermal fibrosis	SSc patients	[18, 20–24]
HSCs	Large improvement in the survival rate Reduction of the disease activity	rp-dcSSc patients	[25]
Autologous HSCs and high-dose CYC	Death of seven patients Progression of interstitial pneumonia	SSc patients	[26]
PB-SCT and high-dose CYC	Improvement in interstitial pneumonia	SSc patients	[20]
HSCs	Improved pulmonary function Improved inspiratory vital capacity	SSc patients	[22]
HSCs	No significant improvement in lung and other organ function	SSc patients	[18, 27, 28]
HSCs	No improvement in immunological markers No improvement in clinical status Death of one patient 2 days after HSCT due to cardiac failure	SSc patients in the advanced stage of disease	[29]
HSCs	No significant improvement in the disease in the short term A large improvement in lung function after 1 year of HSCT	SSc-ILD patients	[30]
HSCs	Increasing overall survival rate Increasing event-free survival rate	Severe SSc patients	[31]
HSCs	Improved function Improved physical capacity Improved quality of life at 1 year after HSCT	SSc patients	[32]
Autologous HSCs	Improvement of physical outcomes Improvement in HAQ-DI score No improvement in the MCS of the SF-36 score	SSc patients	[33, 34]
HSCs	No change in vessel density Reduction in the mRSS Significant decrease in histological scores of skin fibrosis	SSc patients	[35]
HSCs and high-dose CYC	Improvement of microvascular remodelling	SSc patients	[4, 36]
HSCs	Improvement in the disease Induction of Th1 response Suppression of Th2 response	TSK/+ mice animal model	[37]
HSCs	Increase in IFN-γ Reduction in IL-4 Reconstitution of CD8^+^ T cells, memory CD4^+^CD45RO^+^ T cells and CD19^+^ B cells No reconstitution of naïve CD45RA^+^ T cells and Tregs as expected Decrease of cytokines, including TNF-α, TGF-β, IL-6 and IL-2R Early recovery of neutrophils and platelets after HSCT	SSc patients	[20, 26, 38]
HSCs	Increase in the serum level of cytokines such as IL-6, IL-8 and IFN-γ Significant decrease in the skin fibrosis	SSc patients	[39]
HSCs	Increased number of total B cells Change in the number of B cell subtypes Decreased number of memory B cells and plasma cells Increase in the number of naïve B cells Increased secretion of IL-10 by naïve B cells Increased frequency of IL-10–producing CD19^+^CD24^hi^CD38^hi^ Bregs as well as CD19^+^CD24^hi^CD27^+^ Bregs Increase in the frequency and suppressive function of CD4^+^CD25^hi^FoxP3^+^ Tregs	SSc patients	[39]
HSCs	Significant decrease in the serum level of anti-Scl-70 autoantibody	SSc patients	[40]
HSCs	Improvement in the number of CD8^+^ T lymphocytes Improvement in the number of NK cells Reduction in neutrophil transcripts	SSc patients	[41]
CD34-selected HSCs	Improvement of the mRSS Improvement in pulmonary function Low complication rate of HSCT 8 years post-transplantation No treatment-related deaths	SSc patients	[31]

## MSCs

MSCs were introduced by Friedenstein *et al.* [42]. The cells appeared like fibroblasts and were derived from the bone marrow (BM). The cells were able to differentiate to fibroblasts, adipocytes, chondrocytes and osteocytes. MSCs, and mesenchymal stromal cells, another type of stem cell, can be found in the other tissues, including umbilical cord MSCs (UC-MSCs), endometrial polyps, menses blood and adipose tissue. These cells express CD73, CD90 and CD105, while they are negative for CD34, CD45, CD14, CD11b, CD79α, CD19 and human leucocyte antigen (HLA)-DR [43–45].

MSC characteristics that give them advantages for the treatment of autoimmune diseases include [46]:

- Isolation and expansion of MSCs is not complex [47].
- MSCs are capable of migration towards injured tissues for repair [48].
- MSCs have low immunogenicity, as they do not express MHC-II and co-stimulatory molecules, including B7-1, B7-2 and CD40. Therefore, they are not recognized by T cells and are not rejected after allogeneic transplantation [43, 49].
- MSCs suppress T cell proliferation and cytokine secretion [50, 51].
- MSCs prevent B cell proliferation and autoantibody production [52].
- MSCs inhibit dendritic cell (DC) maturation and prevent NK cell cytotoxicity [53, 54].

Notably, the multiplication power and differentiation capacity of MSCs vary and depend on the type of tissue from which MSCs originated. For example, UC-MSCs have higher capacities in multiplication power and differentiation to various cells compared with BM-MSCs [55].

## HSCs

HSCs are pluripotent stem cells that differentiate into multiple blood cells. HSCs are commonly characterized by the absence of lineage-specific markers of blood cells. Human HSCs are detected as CD34^+^, CD59^+^, CD90/Thy1^+^, CD38^low/−^, c-Kit^−/low^ and Lin^−^. Mouse HSCs are considered as CD34^low/−^, SCA-1^+^, Thy1^+/low^, CD38^+^, c-Kit^+^ and Lin^−^ [56, 57].

HSCT was first introduced in 1950 in a mouse model of leukaemia/lymphoma. Allogeneic injection of healthy BM to mice as well as an X-ray conditioning regimen increased the survival of mice. Thomas *et al.* [58] performed the first HSCTs in children and adults with leukaemia that had promising results.

HSCT, i.e. bone marrow transplantation (BMT), is now used as a treatment for malignant diseases such as leukaemia, myeloma and lymphoma, as well as non-malignant diseases such as immunodeficiency disorders, haemoglobinopathy and autoimmune rheumatic diseases. MSCT or HSCT could be an effective therapeutic option in autoimmune rheumatic disease patients that are resistant to conventional therapies (refractory autoimmune diseases) [59].

In autoimmune diseases, HSCT helps to re-establish immune system tolerance after removal of autoreactive memory cells through conditioning regimens. Indeed, the adaptive immune system is readjusted so that it does not recognize self-antigens and is tolerant to self-antigens [60].

## MSC transplantation in human SSc patients

The clinical course of the disease improves after MSCT, as well as an improvement in the modified Rodnan skin score (mRSS) and HAQ disability index (HAQ-DI) score [10, 61].

Christopeit *et al.* [12] showed MSCs from SSc patients have immunosuppressive features; however, they have defects in the differentiation capabilities, such as differentiation to endothelial progenitor cells that contribute to vasculogenesis. In addition, BM-MSCs from SSc patients express more TGF-β receptor ІI compared with MSCs from healthy people. This leads to the higher activation of the suppressor of mothers against decapentaplegic (SMAD) pathway, leading to more collagen 1α2 synthesis. Therefore, autologous MSCT may not be appropriate for the treatment of SSc patients and the donors of MSCs should be chosen carefully (allogenic MSCs *vs* autologous MSCs) [62]. Moreover, *Akiyama et al.* [63] showed that MSCs from SSc patients express lower levels of FAS and FAS ligand compared with healthy people. Therefore, they have a lower capacity in the induction of apoptosis in T cells. On the other hand, comparison of BM-MSC characteristics between SSc patients and healthy people showed no significant differences in phenotype, proliferation rate, differentiation capability and immunosuppression features [64]. This controversy may be explained by the severity of SSc disease, as MSCs from SSc patients with different severity may show distinct characteristics. For example, it has been shown that MSCs from patients with early severe rapidly progressive diffuse SSc (rp-dcSSc) overexpress bioactive mediators that lead to inflammatory response as well as pro-angiogenic growth factors [13]. Therefore, they may contribute to progression of the disease, suggesting autologous MSCs from SSc patients in the advanced stage of disease are not suitable candidates for MSCT. Considering these controversies, more studies are needed.

Allogeneic MSCT in a female patient with SSc had promising results, and an improvement in skin score as well as pain score was observed. The results showed ligation of CD137L with its receptor was effective in immunomodulation by induction of type 1 cell-mediated immune response in the patient, ameliorating the disease [12]. Digital ulcers are a common and debilitating ischaemic manifestation in SSc and represent end-organ damage from progressive vasculopathy. They serve as a marker of disease severity and internal organ involvement [65]. Evaluation of the effect of allogeneic MSCT on digital ulcers showed MSCs have vasculoregenerative features and show immunomodulatory properties. These lead to an improvement in digital ulcers observed in 30–50% of patients [14]. Therefore, MSCs could be useful in the improvement of angiogenesis and neovascularization in SSc patients; however, more studies are needed to address this issue. Despite the advice for avoidance of autologous MSCT in SSc patients, results of autologous MSCTs in a female SSc patient with critical limb ischaemia showed accelerated vascular network recovery, restoration of blood flow and a reduction of skin necrosis [13].

Combined therapy, including allogeneic MSCT, and plasmapheresis was accompanied by an improvement in the mRSS and lung function. The mRSS measures skin thickness in SSc patients and is used to evaluate the efficacy of treatment. In addition, a decrease in anti-Scl-70 autoantibody titres, serum TGF-β and VEGF was detected. No adverse effect was reported with this therapy [10]. These results suggest a combination consisting of MSCT and other therapeutics may be effective or more effective than MSCT alone for the treatment of SSc.

## HSCT in SSc

Considering that SSc is an autoimmune disease, a non-myeloablative conditioning regimen consisting of anti-thymocyte globulin or anti-CD52 and CYC can be effective in removing autoreactive immune cells before transplantation [66]. It has been shown that a myeloablative conditioning regimen, including total body irradiation (TBI), as well as CYC or busulfan, could cause mortality, treatment-related deterioration of internal organ (lung and kidney) function and radiation-related myelodysplastic syndrome/leukaemia [18]. To reduce these side effects, most centres use a non-myeloablative conditioning regimen in SSc patients before HSCT.

In SSc patients with high cardiac involvement that have a contraindication for a high dose of CYC (200 mg/kg), a fludarabine-based regimen is used as a conditioning regimen that is relatively safe, with a transplant-related mortality of 2.4% and a neutropenic interval of 5.2 days [67].

Clements *et al.* [68] suggested autologous HSCT could be beneficial in patients with diffuse SSc who have had the disease for a short duration (<3 years from the first non-RP symptom) and mild involvement of the heart, lung or kidney. In contrast, they found that autologous HSCT is not a good therapeutic option in SSc patients with severe or end-stage disease in which the organs are highly involved.

### Evaluation of HSCT efficacy based on survival rate and skin involvement in SSc patients

Results of several studies have shown that HSCT is a safe and feasible therapeutic method in SSc patients [20, 25, 27]. In addition, it is associated with increased survival and complete or partial remission [15, 16, 20, 27, 28]. Long-term follow-up of SSc patients who received HSCT showed that 82% of patients had a good response. CYC was used as a conditioning regimen in one study [15].

Evaluation of HSCT in patients with SSc showed survival of 96.2% after 5 years and 84.8% after 7 years. In addition, event-free survival (disease-free survival) of 64.3% after 5 years and 57.1% at 7 years after HSCT was reported [15]. In another study where a combination of HSCT and CYC (i.v. injection) was used for the treatment of SSc patients, survival of 93% for both 5 and 10 years after transplantation and event-free survival of 50% at 5 years and 40% at 10 years was reported [16]. A combination of HSCT and high-dose CYC in early dcSSc resulted in better long-term event-free survival and an acceptable survival rate compared with i.v. CYC alone; however, the treatment was associated with early death in some SSc patients [17]. In another study, Oyama *et al.* [18] reported that after 25.5 months of follow-up of SSc patients after HSCT, survival and disease-free survival rates were 90% and 70%, respectively. Altogether, these results suggest that HSCT has promising survival and event-free survival rates.

HSCT in a group of SSc patients resulted in skin improvement and a significant decrease in the mRSS [19]. In another study, a combination of high-dose CYC and peripheral blood stem cell transplantation (PB-SCT) led to a significant decrease in the skin score in all SSc patients. In addition, biopsy of the skin after PB-SCT showed a decrease in dermal fibrosis [15, 16, 18, 20–24]. An efficacy comparison of selected CD34^−^ stem cells and unselected CD34^+^ stem cells for HSCT showed no difference between the two groups in the improvement of skin score. This indicates stem cell manipulation does not improve skin involvement after HSCT in SSc patients [69].

The efficacies of HSCT and conventional therapy in SSc patients were compared in two groups of patients with rp-dcSSc. rp-dcSSc is associated with severe internal organ involvement and high mortality. While HSCT caused great improvement in the survival rate of the patients and was effective in the reduction of the disease activity, the therapeutic efficacy of conventional therapy was not as promising as HSCT. This suggests HSCT may be more effective than conventional drugs in the treatment of rp-dcSSc patients [25]. On the other hand, administration of a combination of high-dose CYC and autologous HSCs to SSc patients led to the death of seven patients due to progression of interstitial pneumonia. This indicates patients with high involvement of organs should be excluded from HSCT [26]. Patients with no severe heart, lung or other internal organ involvement could be promising candidates for HSCT.

### Evaluation of HSCT efficacy based on organ function in SSc patients

SSc affects organs and organ function, including the heart, lung, kidney and gastrointestinal tract [70]. Vonk *et al.* [15] reported a sustained positive effect of HSCT on organ function. High-dose CYC as well as PB-SCT resulted in an improvement in interstitial pneumonia [20]. Also, HSCT improved pulmonary function and inspiratory vital capacity of SSc patients [22]. On the other hand, HSCT did not produce a significant improvement in lung and other organ functions [18, 27, 28]. In a study of three SSc patients in the advanced stage of the disease, two of three patients did not respond to HSCT, as immunological markers and clinical status did not improve. In addition, one of three patients died after 2 days of HSCT due to cardiac failure [29].

A reduction of vessel density in SSc patients results in a reduction of capillary blood flow and the subsequent lack of nutrient delivery to the cells and hypoxia of the tissues [64]. The effect of HSCT on skin vessel density was examined by CD31^+^ staining, von Willebrand factor (VWF) staining and VE-cadherin staining in the superficial and deeper dermis. Although no change in vessel density was detected, a reduction in the mRSS and a significant decrease in histological scores of fibrosis were observed after HSCT [35]. In SSc, vascular and microvascular structures are damaged, contributing to the onset and development of digital ulcers and PAH [71]. In one study, the therapeutic efficiency of a combination of HSCT and high-dose CYC was compared with a combination of HSCT and low-dose CYC in the improvement of microvascular remodelling. Results of the study showed the combination of HSCT and high-dose CYC was more effective in microvascular remodelling [4, 36].

Ciaffi *et al.* [30] evaluated the therapeutic efficacy of HSCT and i.v. CYC (conventional therapy) in SSc-ILD patients. Results of high-resolution CT, a technique that is especially useful for diagnosing ILD, showed HSCT did not produce a significant improvement in the disease over a short time, however, great improvement in lung function was detected after 1 year of treatment. Moreover, CYC alone did not show a significant improvement in the disease. Additionally, the therapeutic effect of HSCT and CYC was compared in severe SSc patients in another study and event-free survival and overall survival were evaluated. The results showed HSCT was more effective in increasing overall survival and event-free survival than CYC alone. Patients received a non-myeloablative conditioning regimen, and HSCT decreased the requirement for DMARDs after transplantation [31]. These results suggest HSCT therapy may be more effective in the treatment of SSc patients, especially those in advanced stages of disease, than conventional therapy.

An efficacy comparison of selected CD34^−^ HSCs and unmanipulated HSCs in the improvement of SSc showed that CD34^+^ stem cells are more effective in the improvement of the mRSS and pulmonary function than unmanipulated ones. In addition, complications of HSCT were lower in the patients who received CD34^+^ stem cells compared with another group after 8 years of HSCT. No treatment-related deaths were reported. These data suggest the safety of CD34^+^ stem cell therapy. High-dose CYC monotherapy was used for as the conditioning regimen in these patients [72].

### Evaluation of HSCT efficacy based on functional outcomes and immunological parameters in SSc patients

To evaluate functional outcomes in SSc patients, various tests are used, including the mRSS, mouth opening, hand grip strength, range of motion, functional ability of the upper limbs (disabilities of the arm, shoulder and hand questionnaire; Cochin Hand Function Scale), 6-min walk test and quality of life [36-item Short Form questionnaire (SF-36)] [32, 73].

Costa-Pereira *et al.* [32] showed hand function, physical capacity and quality of life of patients significantly improved the first year after HSCT. In another study, the superiority of autologous HSCT *vs* conventional therapy in improvement of physical outcome, was demonstrated. The results showed the HAQ-DI score was significantly improved in the group receiving autologous HSCT, however, the mental component summary of the SF-36 score was not significantly different between the two groups [33, 34]. These results suggest HSCT could improve physical functions of SSc patients.

HSCT is associated with changes in immune system parameters in animal models of SSc. HSCT in TSK/+ mice led to induction of the Th1 response and suppression of the Th2 response. This immunomodulation was associated with improvement in the disease [37]. It has been reported that a shift in Th2 response to Th1 is associated with an amelioration of the skin fibrosis of SSc patients [74]. Evaluation of immunological parameters by Tsukato *et al.* showed HSCT resulted in an increase in IFN-γ and a reduction of IL-4 [26]. These results suggest a shift from Th2 to Th1 contributes to improvement of SSc. A possible explanation for the therapeutic function of IFN-γ may be related to its role in the reduction of collagen synthesis by scleroderma-derived fibroblasts that ultimately contributes to disease improvement. In addition, a change in the blood lymphocytes occurred after HSCT. Reconstitution of CD8^+^ T cells, memory CD4^+^CD45RO^+^ T cells and CD19^+^ B cells was promising, however, naïve CD45RA^+^ T cells and regulatory T cells were not reconstituted as expected. HSCT also decreased the serum level of cytokines, including TNF-α, TGF-β, IL-6 and IL-2R, while all these cytokines showed a tendency towards an increase in serum of SSc patients. Neutrophils and platelets were recovered early after HSCT [26, 75, 76].

Increased levels of serum cytokines, including IL-6, IL-8, IFN-γ and monocyte chemoattractant protein 1 (MCP-1) in SSc patients indicates the presence of an inflammatory milieu in these patients. The Th2 immune response has an undeniable role in the progression of SSc. Interestingly, HSCT increased the serum level of all these cytokines except MCP-1, accompanied by a significant regression in clinical skin fibrosis. These results demonstrate a higher level of inflammatory cytokines than Th2 cytokines contribute to the regression of SSc disease [39].

Another study investigated B cell reconstitution in six SSc patients after 16 months of HSCT. The results showed the number of total B cells increased and a change in the number of B cell subtypes occurred. The number of memory B cells and plasma cells decreased, while an increase in naïve B cells was detected. In addition, secretion of IL-10 by naïve B cells increased. This suggests naïve B cells may improve the disease by production of anti-inflammatory cytokines [24]. Accordingly, the frequency of IL-10–producing CD19^+^CD24^hi^CD38^hi^ regulatory B cells (Bregs) as well as CD19^+^CD24^hi^CD27^+^ Bregs also increased. Bregs suppress immune responses by various mechanisms, such as the production of IL-10. These changes show elimination of self-reactive memory and plasma cells and generation of naïve B cells that are not self-reactive and Bregs contribute to the suppression of immune response by HSCs. HSCs also increased the frequency and suppressive function of CD4^+^CD25^high^FoxP3^+^ Tregs, while SSc patients have a reduction in the number of Tregs [77]. Accordingly, results of another study showed a significant reduction in the serum level of an autoantibody called anti-Scl-70 after HSCT [26]. Anti-Scl-70 autoantibody is seen in 20–35% of patients with SSc and is correlated with poor prognosis of the disease and skin and pulmonary fibrosis [40]. SSc patients show a reduction in CD8^+^ T lymphocytes and NK cells and an elevation in neutrophil transcripts. Comparison of HSCT and conventional therapy showed HSCT is more successful in adjustment of these parameters [41].

### HSCT complications in SSc patients

Complications of autologous HSCTs for SSc patients include acute kidney injury (AKI), treatment-related death, cancers, infections and immunological disorders. Due to the involvement of several organs in SSc disease and high-dose immunosuppressive therapy in the disease, HSCT complications could be more severe than in other autoimmune diseases [28].

HSCT in SSc patients increases the risk of AKI. However, renal shielding, control of blood pressure and avoidance of glucocorticoids could help to reduce AKI in SSc patients undergoing autologous or allogeneic HSCT [61, 78]. Chemotherapy and TBI increase the risk of cancer in these patients [79].

Infections can cause death in SSc patients after HSCT. Results of a study showed cytomegalovirus is the predominant infection that occurs after HSCT, especially in patients with low B cell numbers [80].

The occurrence of secondary autoimmune diseases (SADs) and gonadal failure after HSCT are the other complications in SSc patients. These complications are more common in early rp-dcSSc patients [16, 81]. Results of the study by Strunz *et al.* [82] showed SAD is higher in patients with a greater reduction in the mRSS after HSCT. Another immunological adverse effect is engraftment syndrome (ES). ES is characterized by a combination of fever, erythrodermatous rash, diarrhoea, diffuse capillary leak and non-cardiogenic pulmonary oedema accompanied by neutrophil recovery after HSCT. Studies have shown that ES is more common in patients with older age and cardiac involvement.

To decrease complications of HSCT in SSc patients, Koetter *et al.* reduced CYC and G-CSF. They also modified the conditioning regimen in SSc patients with cardiac involvement. This modified regimen significantly improved their disease and decreased HSCT complications, including a decrease in transplant-related mortality and treatment-related mortality to 4%, and 11%, respectively [22]. In addition, to decrease complications of HSCT, patients should be chosen carefully. Studies show a number of risk factors, including male sex, a low left ventricular ejection fraction (a measure of heart function) and older age, increasing HSCT complications, especially major organ failure and death [83]. Notably, a myeloablative conditioning regimen (TBI) and CD34^−^ selected autologous HSCT had a better long-term outcome compared with CYC in severe SSc patients. However, exposure to TBI is associated with greater haematopoietic toxicity and a risk of secondary cancers [31].

## Conclusion

These results suggest both MSCT and HSCT could improve disease activity and severity in SSc patients. Both treatments resulted in an improvement in the clinical manifestations of the disease and skewed immunological parameters in favour of disease improvement. Overall, both therapeutic methods are safe and few complications have been reported. Engineered MSCs/HSCs expressing various factors such as anti-inflammatory cytokines or combination therapy utilizing MSCs/HSCs and other factors may be more effective in the modulation of immune responses and improvement of the disease. Despite the clear efficacy of MSCT/HSCT in the treatment of SSc shown in the studies, some issues, including the choice of tissue from which stem cells should be derived, autologous *vs* allogenic or xenogeneic origin of stem cells and the type of stem cells (such as MSCs or HSCs), the predictive factors of transplantation outcome remain largely unknown and should be addressed in the future studies to increase the therapeutic efficacy of MSCT/HSCT and minimize the complications of the transplantation.

## Data Availability

No new data were generated in support of this article.
